# Ureter injury in obstetric hysterectomy with placenta accreta spectrum: Case report^☆^

**DOI:** 10.1016/j.ijscr.2021.106489

**Published:** 2021-10-06

**Authors:** Suskhan Djusad, Mohammad Adya Firmansha Dilmy, Arresta Vitasatria Suastika, Raden Muhammad Ali Fadhly, Yuditiya Purwosunu

**Affiliations:** Department of Obstetrics and Gynecology, University of Indonesia, Jakarta, Indonesia

**Keywords:** Placenta accreta spectrum, Subtotal hysterectomy, Ureter injury, Case report

## Abstract

**Introduction and importance:**

Placenta accreta spectrum (PAS) is a state of abnormal attachment of the placenta, including placenta accreta, placenta increta, and placenta percreta. This condition can be life-threatening due to the placenta cannot spontaneously separated, resulting in continuous bleeding. Cesarean section followed by hysterectomy is one of the treatment options for PAS. There was a great liability for urinary tract injuries during the operation of PAS patient.

**Case presentation:**

We present the case of ureter injury during subtotal hysterectomy in patient with PAS. A 30-years-old female patient was diagnosed with recurrent antepartum hemorrhage due to placenta previa accreta spectrum on G2P1 33 weeks of gestational age, singleton live breech presentation, previous c-section 1×. After uterine transverse incision, the baby was delivered. We decided to perform subtotal hysterectomy. There was severe adhesion. On the exploration after subtotal hysterectomy was performed, we found ruptured of the right ureter.

**Clinical discussion:**

Hysterectomy peripartum is one of the treatment of PAS, either to prevent or to control postpartum hemorrhage. In pregnant women with morbid placental adherence, there was a great liability for urinary tract injuries. Distal ureters are the most commonly injured while hysterectomy. Injuries to the ureters in this patient occurred due to severe adhesions and unclear visual organ.

**Conclusion:**

Although it is rare, ureter injury may occur during subtotal hysterectomy in patient with placenta accreta spectrum. To prevent that condition, inserting ureter stent can be perform before the operation. Multidisciplinary approach is carried out so that patient outcomes are good.

## Highlights

• Ureter injury in obstetrics hysterectomy: case report
• Ureter injury in placenta accreta spectrum
• Ureter injury in obstetric hysterectomy with placenta accreta spectrum

## 1 Introduction and importance

Placenta accreta spectrum is general term used to describe abnormal trophoblast invasion into the myometrium of the uterine wall that includes: placenta accreta, placenta increta, and placenta percreta [1]. The incidence of PAS is placenta accreta 63%, placenta increta 15%, and placenta percreta 22% [2]. This condition can be life-threatening and usually necessitates hysterectomy due to the placenta cannot spontaneously separated resulting in continuous bleeding. This case report has been reported in line with the SCARE 2020 guideline [3].

## 2 Patient information

A 30-years-old female pregnant came to the hospital with chief complaint recurrent vaginal bleeding. Patient admitted 32 weeks of gestation. This is her second pregnancy. Her first son was born 7 years ago with previous cesarean section due to preterm rupture of membrane. Patient had antenatal care 5 times. There was no history of vaginal discharge, dysuria, gingivitis. The patient works as a housewife. History of smoking was denied.

## 3 Clinical finding

From physical examination, vital signs within normal limits. Fundal height 26 cm with irregular contraction, fetal heart rate 155 beats per minutes. From speculum examination, there was vaginal bleeding.

## 4 Diagnostic assessment

**Fig. 1:**
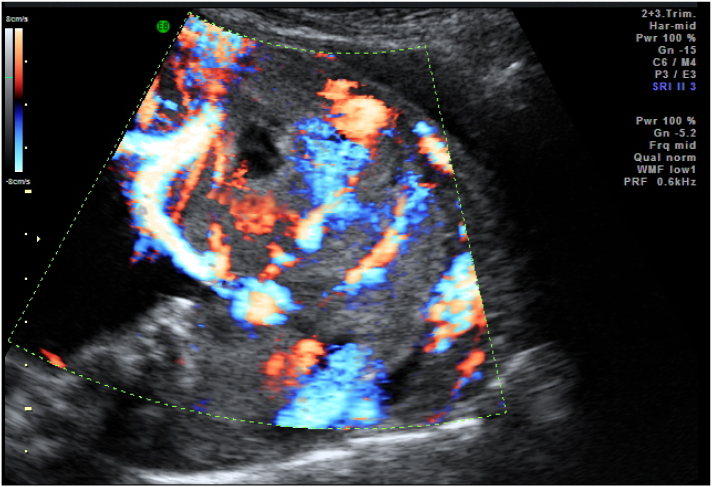
Placenta accreta spectrum ultrasound.

Patient diagnosed with placenta accreta spectrum based on ultrasound finding. We found bridging vessels, lacuna grade III, loss of clear zone, and turbulence flow. The placenta accreta index score was 5 with 69% probability of invasion (Fig. 1).

## 5 Therapeutic intervention

**Fig. 2:**
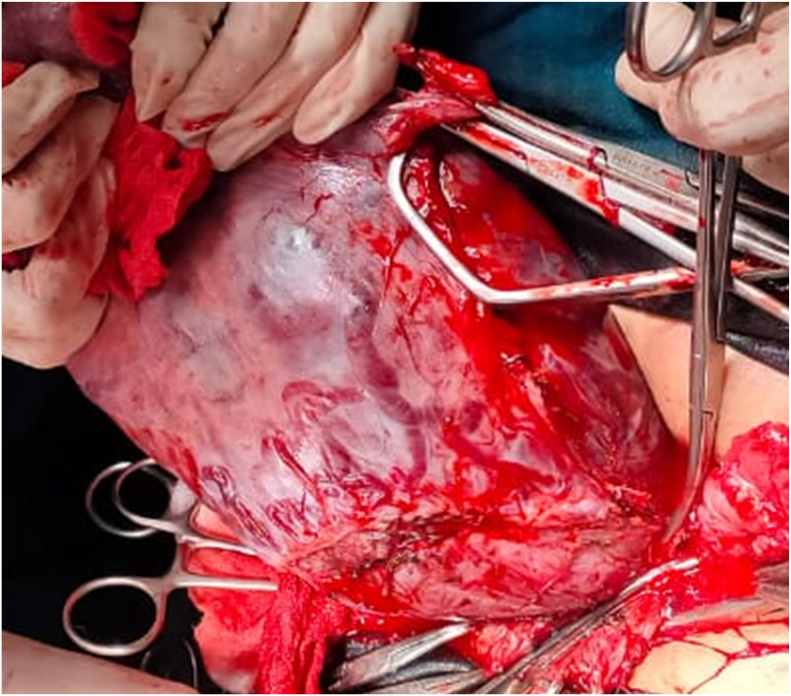
Performing subtotal hysterectomy.

Patient had lung maturation with corticosteroid before the operation. Patient underwent operation with general anesthesia. The operation was performed by maternal fetal medicine (obstetric gynecology). After peritoneum was opened, we found gravid uterus with bluish appearance at right until posterior corpus with enlarge blood vessels (Fig. 2).

We performed transverse incision above placenta edge. After baby was delivered, umbilical cord was clamped, cut, and ligated. Placenta was in situ. Uterine incision was sutured continuously 1 layer using PGA no. 1. We decided to perform hysterectomy. Both round ligaments were clamped, cut, and sutured. Windowing of posterior broad ligaments both tubes and proper ovarian ligaments were clamped, cut, and sutured.

**Fig. 3:**
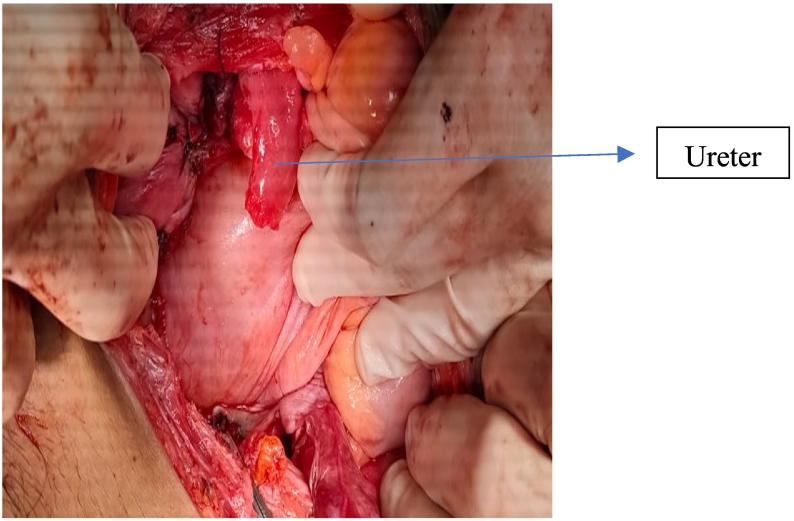
Rupture of right ureter.

There was severe adhesion between lower uterine segment and bladder. We were difficult to identify the ureter. Vesicouterine fold meticulously dissected, bladder was reflected downwards. Both uterine vessels were clamped, cut, and sutured. Both sacrouterine and cardinal ligaments were clamped, cut, sutured. Uterus was cut as the portio. Cervical stump was sutured using PGA no. 1 (Fig. 3).

**Fig. 4:**
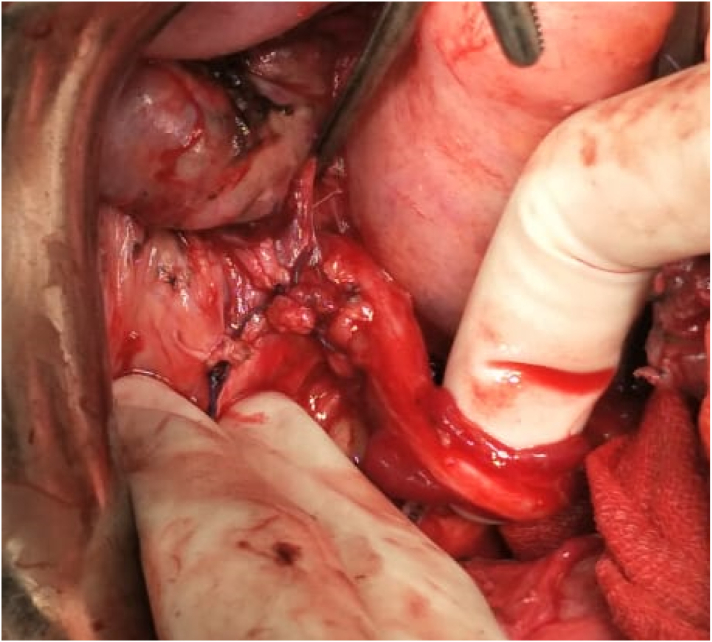
Post reimplantation of right ureter.

On the exploration, we found rupture of right ureter. The operation continued by urologist with reimplantation of right ureter and DJ stent insertion procedure. We ensure there was no active bleeding. Abdominal wall closed layer by layer. Intraoperative bleeding was 1400 ml (Fig. 4).

## 6 Follow up and outcome

Post operations day 3, vital sign within normal limit and the patient was discharge in satisfactory condition. From histopathology result, specimen correspond to placenta accreta spectrum. She is being followed up 1 weeks postoperative and admitted spontaneous micturition. Patient and their families are satisfied with the performance and services of doctors dan hospital.

## 7 Discussion

Placenta accreta spectrum (PAS) is general term used to describe abnormal trophoblast invasion into the myometrium of the uterine wall [1]. Patient had previous c-section and low lying placenta, which is the most important risk factor for development of PAS. Defective deciduallization (thin, poorly formed, partial, absent, or dysfunctional decidua) is the most common theory of PAS. Previous uterine surgery causes scarring of the endometrial-myometrial interface and allows the anchoring of the placenta attach directly to or invade the myometrium [4], [5]. Based on a multivariate analysis, placenta previa appeared to be an independent risk factors for PAS (odds ratio 54, 95% CI 18-166), while prior uterine surgery was not (OR 1.5, 95% CI 0.4-5.1) [6]. The frequency of PAS increased with an increasing number of cesarean deliveries. Primary cesarean birth increased 3% the frequency of PAS [7]. From history taking, except previous cesarean surgery, patient did not have other risk factors of PAS such as history of uterine surgery (myomectomy, hysteroscopic, dilation and curettage), manual removal of placenta, postpartum endometritis, infertility procedures (e.g. transfer of cryopreserved embryos), multiple gestation.

The clinical manifestation of PAS is bleeding while trying to detach the placenta. Wherein the placenta remains attached to the myometrium. It is also may present as antenatal bleeding. This is in accordance with the patient's complain that she admitted recurrent bleeding before at term.

Based on prenatal ultrasound staging system for placenta accreta spectrum (PAS) disorders and corresponding histopathological findings and grade of PAS disorder, the patient categorized PAS 1. This finding based on the presence of placental lacunae, loss of clear zone, and the histopathology was placenta accreta.

The best time for schedule delivery is still controversial [1]. The risk of bleeding can occur if waiting for term gestation and causing emergency delivery. Performing emergency operation causing suboptimal condition for the patient and baby. On the other hand, there is a risk of pretem birth if the baby was delivered before term to prevent the bleeding. Patient complained recurrent antepartum hemorrhage. Therefore, after lung maturation, we performed cesarean section. Corticosteroids is recommended for pregnant women between 24 0/7 weeks and 33 6/7 weeks of gestation, who are at risk of preterm delivery [8].

Hysterectomy peripartum is one of the treatment of PAS, either to prevent or to control postpartum hemorrhage. In systematic review including 7001 PAS cases, peripartum hysterectomy was performed in 52.2% (95% CI 38.3–66.4), and blood transfusion was required in 46.9% (95% CI 34.0–59.9) [2].

In pregnant women with morbid placental adherence, there was a great liability for urinary tract injuries. Alanwar et al [9], stated the incidence of ureter injury was 4.7%. Distal ureters are the most commonly injured while hysterectomy. Injuries to the ureters in this patient occurred due to severe adhesions and unclear visual organ. While the operation is performed in a tertiary hospital, urologist should we call to repair the injury of ureter.

Multidisciplinary approach with urologist should be done to reduce the possibility of ureter injuries by inserting ureter stent. Preoperative ureteral catheterization is recommended in cases of parametrial invasion of the uterus.

## 8 Conclusion

Hysterectomy is one of the PAS treatment options. Although it is rare, ureter injury may occur during surgery. To prevent that condition, inserting ureter stent can be perform before the operation. Multidisciplinary approach is carried out so that patient outcomes are good.

## Funding

No funding sources for the publication of this article.

## Ethical approval

This study is exempt from ethical approval in our institution.

## Consent

Written informed consent was obtained from the patient for publication of this case report and accompanying images. A copy of the written consent is available for review by the Editor-in-Chief of this journal on request.

## Research registration number

Not applicable.

## Guarantor

Suskhan Djusad MD.

Mohammad Dilmy MD.

Raden M Ali Fadhly MD.

## Provenance and peer review

Not commissioned, externally peer-reviewed.

## CRediT authorship contribution statement

Suskhan Djusad: concept, revising, final approval.

Mohammad Dilmy: operator, editor.

Arresta V Suastika: data collection.

Raden M Ali Fadhly: drafting, data collection, writing.

Yuditiya Purwosunu: operator.

## Declaration of competing interest

The authors declared no conflict of interest.
